# Bioregulatory periodontal medicine: could the molecular networks, inflammation and system biology modify the auto-regulatory capacity of the patients?

**DOI:** 10.3389/fphys.2026.1799419

**Published:** 2026-09-14

**Authors:** María Carolina Varas-Reyes, Bertha Arelly Carrillo-Ávila, Victor Martínez-Aguilar, Marco Antonio Álvarez-Pérez, Jaime Díaz-Zúñiga, Samanta Melgar-Rodríguez

**Affiliations:** 1 Conservative Dentistry Department, Faculty of Dentistry, University of Chile, Santiago, Chile; 2 Faculty of Dentistry, Autonomous University of Yucatán, Mérida, Mexico; 3 Tissue Bioengineering Laboratory, División de Estudios de Posgrado e Investigación (DEPeI), Faculty of Dentistry, National Autonomous University of Mexico, Mexico City, Mexico; 4 Department of Medicine, Faculty of Medicine, University of Atacama, Copiapó, Chile

**Keywords:** auto-regulation, bioregulatory system, chronic diseases, integrative medicine, regulatory response

## Abstract

**Background:**

The traditional healthcare model, focused on symptom management, has shown limitations in addressing chronic diseases. In response, Bioregulatory Systems Medicine (BrSM) has emerged, an approach that promotes the body’s self-regulation through multi-component drug and phytotherapeutic treatments. Its potential application in dentistry, particularly in periodontitis, has spurred the study of various natural molecules with the potential to prevent or reduce bone resorption. This qualitative systematic review analyzes the available evidence from animal and human models in the field of bioregulatory periodontal medicine.

**Methods:**

A qualitative systematic review was conducted according to PRISMA-P guidelines. Animal and human studies published in English or Spanish within the last 10 years were included. The search was performed in five databases (SciELO, CENTRAL, PubMed, LILACS, and Web of Science) using MeSH terms. Data selection and analysis were performed in duplicate. Tools were applied to assess the risk of bias: SYRCLE for animal studies, Newcastle-Ottawa for observational studies, and RoB 2 for randomized controlled trials.

**Results:**

A total of 1,252 articles on calendula, caffeine, piperine, curcumin, and omega-3 fatty acids were found. Fifty-three studies were selected based on inclusion and exclusion criteria. Calendula and piperine reduced bone loss and inflammation in animal models. Curcumin and omega-3 fatty acids showed these effects in both animals and humans. For caffeine, the evidence was contradictory, although antibacterial activity was observed in experimental models.

**Conclusion:**

Bioregulatory molecules decrease or prevent bone resorption caused by periodontitis, both in experimental animal studies and in clinical trials in humans.

**System Review Registration:**

https://osf.io/, identifier 3WHNA.

## Introduction

The prevailing healthcare model is rooted in a reductionist biomedical approach widely recognized in 2026. In the reductionist paradigm the disease is treated as a mechanical dysfunction of parts rather than a disorder of the whole person (Wade and Halligan, 2004; De Vreese et al., 2010; Hayward, 2019). Healthcare professionals often specialize deeply in body systems or tissues, and focus on diagnosing and treating specific diseases by identifying symptoms. Nowadays, the shift from a purely biomedical model to a biopsychosocial model represents a significant transformation in how modern care is delivered, and patient outcomes have improved by combining medical treatment with emotional support, team-based coordination, and continuous quality improvement (Wade and Halligan, 2004; Goldman et al., 2015; Hayward, 2019; Alsaqer et al., 2025). Bioregulatory Systems Medicine (BrSM) is a new medical paradigm that views the human body as a complex organism, comprised of physiological networks: molecular, cellular, tissue, organ, system, and individual. At each of these levels, different actors—molecular, cellular, or tissue—maintain or restore the individual’s homeodynamics (Goldman et al., 2015). This concept is known as auto-regulatory capacity, which encompasses both individual networks and the interactions between them, forming a complex system to study (Goldman et al., 2015). From this perspective, disease is viewed as a network disruption, causing instability and loss of normal body function (Fioranelli et al., 2019). The conceptual core of BrSM focuses on modulating network dysregulations and enhancing intrinsic self-regulation to restore physiological homeodynamics or establish a new stable equilibrium (Fioranelli et al., 2019).

The BrSM proposes that these effects can be achieved through the use of low-dose medications with precise, targeted, and synergistic bioregulatory capabilities. These medications are composed of different therapeutic components (multicomponent) with effects on different targets (multitarget) and a favorable safety profile (Goldman et al., 2015; Makhoba et al., 2020). In this way, a more comprehensive and systematic approach to patient care is sought, based on a thorough evaluation of their medical history, recognition of their specific characteristics, and the stage of disease progression, often called patient/person-centered care (Fioranelli et al., 2019). This concept moves beyond just treating a disease to understanding the entire individual (biology, biography, context) for better diagnosis and tailored treatment plans, integrating history, unique traits, and disease stage for comprehensive management (Saha et al., 2008). It is in this context that periodontitis —considered a chronic non-communicable disease— emerges as a disease that could be treated from this perspective (Papapanou et al., 2018).

Periodontitis is a chronic non-communicable disease (CNCD) caused by dysbiosis of the subgingival microbiota and is characterized by the progressive destruction of the tooth’s supporting tissues, which without treatment leads to tooth loss (Hajishengallis et al., 2012; Hajishengallis, 2014; Hajishengallis, 2015; Papapanou et al., 2018). Its treatment is symptomatic: where destruction is present, scaling and root planing are performed (Lamont et al., 2018). Although mathematical models exist to determine individual risk and schedule follow-up appointments, there is no definitive cure, therefore, patients must learn to recognize the signs of disease activity to prevent recurrence and progression (Khumbudzo et al., 2025). Furthermore, periodontitis can trigger a chronic low-grade inflammatory phenotype (CLIP). In fact, even after successful periodontal therapy, evidence shows that CLIP persists for at least six months after a clinical successful treatment (Zekeridou et al., 2019). This demonstrates that the effects of periodontitis on other organs or tissues persist even when the patient is considered “healthy.” In recent years various natural components including calendula, caffeine, piperine, curcumin, and ω-3 fatty acids, among others, have been investigated for their effects on modulating periodontitis associate bone resorption and subsequent bone loss (Alexandre et al., 2017; Astaneh et al., 2024; Abdelnabi and Mohsin, 2025; Liu and Chai, 2025).

Given that BrSM promotes treatments based on the body’s auto-regulation and the use of natural compounds with multiple therapeutic targets, questions arise regarding its applicability in dentistry. Within this framework, several phytotherapeutic substances have been identified with the potential to reduce bone resorption through various cellular or molecular mechanisms. Periodontitis, due to its chronic inflammatory nature and its association with progressive and irreversible bone resorption, is presented as a CNCD that can be addressed from this therapeutic perspective. Thus, the following question arises, which this systematic review aims to answer: Are bioregulatory molecules capable of reducing or preventing periodontitis-induced bone resorption in animal experimental models or humans?

## Methods

The following qualitative systematic review was conducted following the PRISMA-P protocol guidelines (Page et al., 2021). The study was based on the PICO question: population (patients with periodontitis or animals affected by periodontitis), intervention or exposure (curcumin, calendula, ω-3, or other), comparison (patients with periodontitis treated with the gold standard and animals with periodontitis not receiving treatment), and outcome measures (reduction or prevention of bone resorption due to periodontitis).

### Eligibility criteria: inclusion and exclusion

The selected studies were experimental studies in animal models and human intervention studies related to the reduction of bone resorption caused by periodontitis through the administration of bioregulatory agents. The selection criteria were articles published within the last 10 years. Studies in spanish were selected because it is the native language of the authors, and english was selected because it is the universal language of science. The inclusion and exclusion criteria applied in the full-text analysis were as follows:

- All studies describing the association between bioregulatory molecules and bone resorption caused by periodontitis in experimental models or human studies were included.
- All studies in languages ​​other than Spanish or English were excluded.

### Search strategy

An electronic search was conducted in the SciELO, Cochrane Library (CENTRAL), Medline via PubMed, LILACS, and Web of Science databases. The search strategy used MeSH terms in the various databases, adapted for this search strategy. The strategy search is found in Appendix S1.

### Studies selection

The selection of studies was performed independently and in duplicate by two reviewers (MCV and JD-Z). In cases where there were doubts regarding the inclusion of a study, a third reviewer was responsible for deciding its inclusion or exclusion (SM-R). The articles retrieved from each database were grouped, and duplicates were subsequently removed. Titles and abstracts were then evaluated, and records unrelated to the review objectives were eliminated. Finally, the studies that met the criteria were downloaded for full-text analysis.

### Data extraction

Data extraction was performed independently and in duplicate by two reviewers (MCV, JD-Z), and a third reviewer verified and confirmed the accuracy of the information (SM-R). When disagreements arose between reviewers, they discussed the matter to reach a consensus, and if the discrepancy persisted, a third reviewer made the final decision (SM-R). Data extraction followed a structured approach; a template was created to extract the key features of each included document (**Supplementary File 2**). The data elements included in the template were the first author’s name, year of publication, study design, study population (number, sex, and age), group definition, intervention or exposure, periodontal diagnostic criteria, results regarding the effect on bone resorption caused by periodontitis, and origin. The Cochrane Handbook was used as a guide for the data collection process (Higgins et al., 2024).

### Outcomes definition

Once the articles were selected, they were read in full, identifying primary, secondary, and tertiary outcomes to complete the respective tables. The primary outcome was defined as the use of bioregulatory molecules to prevent bone resorption in experimental models of periodontitis or in clinical studies. The secondary outcome was defined as the application of bioregulatory molecules in the treatment of periodontitis, both in experimental models and in studies conducted in humans. Finally, the tertiary outcome included all studies that defined or conceptualized the terms periodontology or bioregulatory dentistry.

### Data analysis

The selected articles were qualitatively assessed to investigate the effect or use of bioregulatory molecules for the prevention or treatment of periodontitis, either in humans or in animal models. The Systematic Review Center for Laboratory Animal Experimentation (SYRCLE) tool was used to assess the risk of bias (RoB) in animal experiments. The Newcastle-Ottawa tool was used for clinical studies, including cohort and case-control studies. Risk of Bias 2 (RoB 2) was applied to randomized controlled trials.

SYRCLE is derived from the Cochrane RoB tool for clinical studies and was adapted for use in animal studies (Hooijmans et al., 2014; Higgins et al., 2024). The tool consists of 10 main questions or domains related to selection bias, performance bias, detection bias, attrition bias, reporting bias, and other biases. Signaling questions were used to support the main questions in order to determine the RoB. Responses to the tool’s questions were given as “Yes” (question adequately answered), “No” (question not answered), or “Unclear” (insufficient information to answer yes or no). Based on these responses, the RoB domains were classified as low, high, or unclear.

To assess the RoB in non-randomized studies, such as observational studies, the Newcastle-Ottawa Scale (NOS) was used. With this tool, each study was evaluated based on nine items, categorized into three groups: the selection of study groups, the comparability of the groups, and the determination of the exposure or outcome of interest. The stars awarded for each quality article serve as a quick visual assessment. A score above 7 stars was considered high quality; between 5 and 7 stars, moderate quality; and less than 5 stars, low quality (Silver et al., 2016).

For randomized controlled trials, the Cochrane RoB 2 tool was used. This tool assesses the RoB in five domains: bias in the randomization process, bias due to deviations from planned interventions, bias due to missing outcome data, bias in outcome measurement, and bias in the selection of the reported outcome. Each domain is rated as “low risk,” “some concern,” or “high risk” of bias, and an overall judgment on the study’s risk of bias is provided (Sterne et al., 2019).

## Search results and discussion

A total of 1,251 articles were retrieved from the PubMed (395), SciELO (110), Cochrane Library CENTRAL (185), LILACS (282), and Web of Science (279) databases during the identification phase. Of these, 621 duplicates were removed, leaving 630 selected for title and abstract review. At this stage, 538 studies were excluded because their objective was not aligned with the research question. Following this, 92 articles were analyzed for eligibility through full-text review, of which 39 were excluded for the reasons described in **Figure 1**. Finally, 53 studies were included in the systematic review, distributed as follows: 3 correspond to studies with Calendula (**Supplementary Table 1A**), 5 with Caffeine (**Supplementary Table 1B**), 2 with piperine (**Supplementary Table 1C**), 23 with Curcumin (**Supplementary Table 1D**) and 20 with ω-3 fatty acids (**Supplementary Table 1E**). The **Supplementary Table 2** identifies the outcome contribution of each selected article.

**Figure 1:**
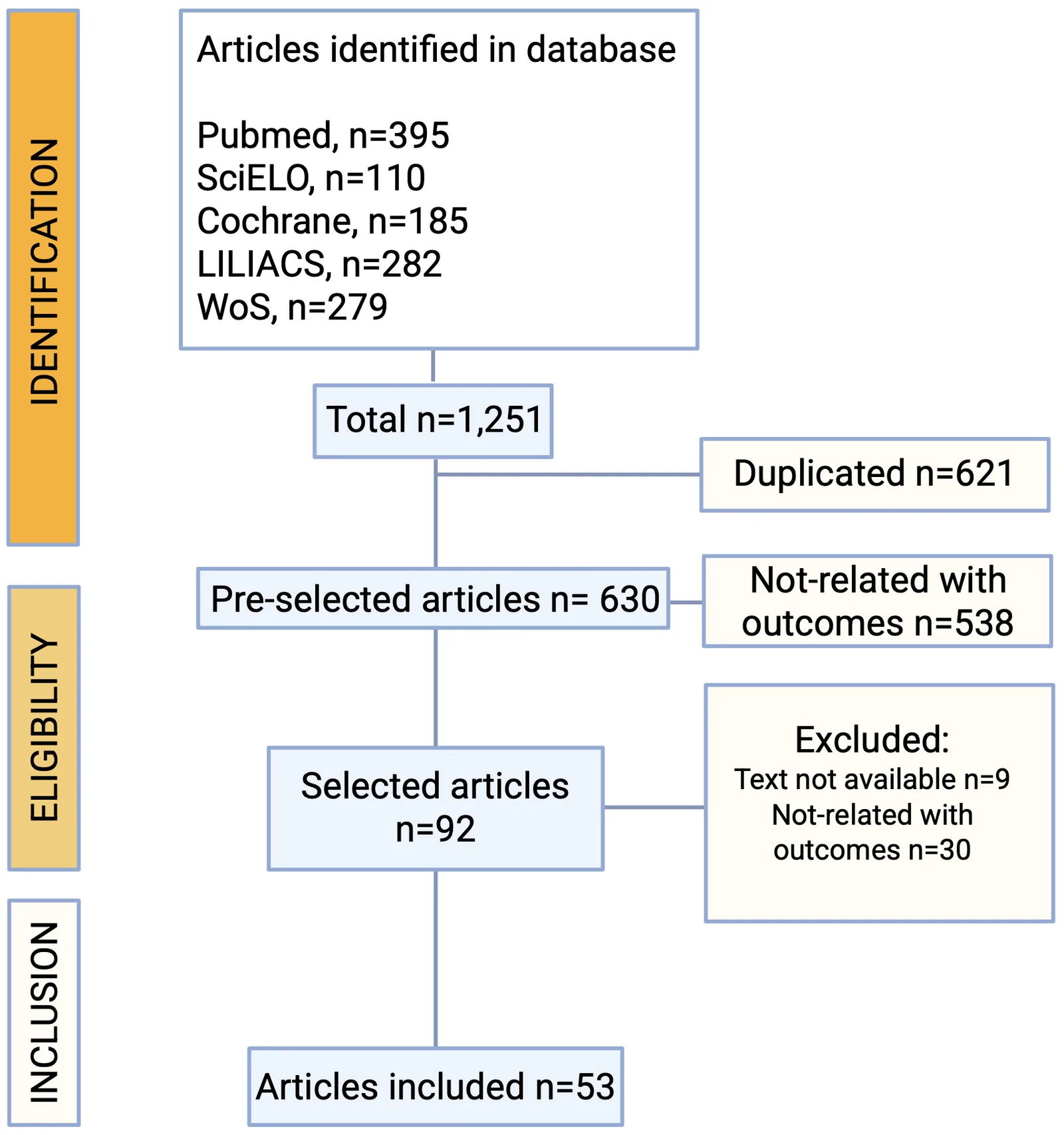
PRISMA flowchart. Summary of the search and selection of studies. From a total of 1,251 articles, duplicates were filtered, leaving 630 studies for abstract and title review. 538 studies were excluded because they were unrelated to the research question or because they were systematic reviews with or without meta-analysis and narrative reviews. Subsequently, 92 full-text articles were reviewed, applying inclusion and exclusion criteria, and 53 articles were ultimately selected.

### Calendula

Calendula (CLO) is not a single compound but a source of several bioactive phytochemicals because it is a flower extract and is overall yellow-orange, lipophilic, and variably soluble in alcohol. Moreover, its major bioactive compounds are triterpenoid esters, such as faradiol monoesters; triterpenoid alcohols, such as faradiol and oleanolic acid; flavonoids, such as quercetin, glycosides, and isorhamnetin; saponins such as calendulosides; carotenoids, such as lutein and β-carotene and zeaxanthin; and volatile oils such as α-cadinol (Kishimoto et al., 2005; Shahane et al., 2023). Structurally, the triterpenoids have a basic structure of 30 carbons arranged in four or five rings; for example, faradiol monoesters are C_30_H_60_O_2_ derivatives esterified with fatty acids; the flavonoids are structures with a C6-C3-C6 skeleton (two aromatic rings joined by a three-carbon bridge), for example, quercetin is C_15_H_10_O_7_ and isorhamnetin is C_16_H_12_O_7_ (**Figures 2A, B**), which impart antioxidant activity (Neukirch et al., 2004; Olennikov et al., 2017; Bilušić et al., 2024).

**Figure 2:**
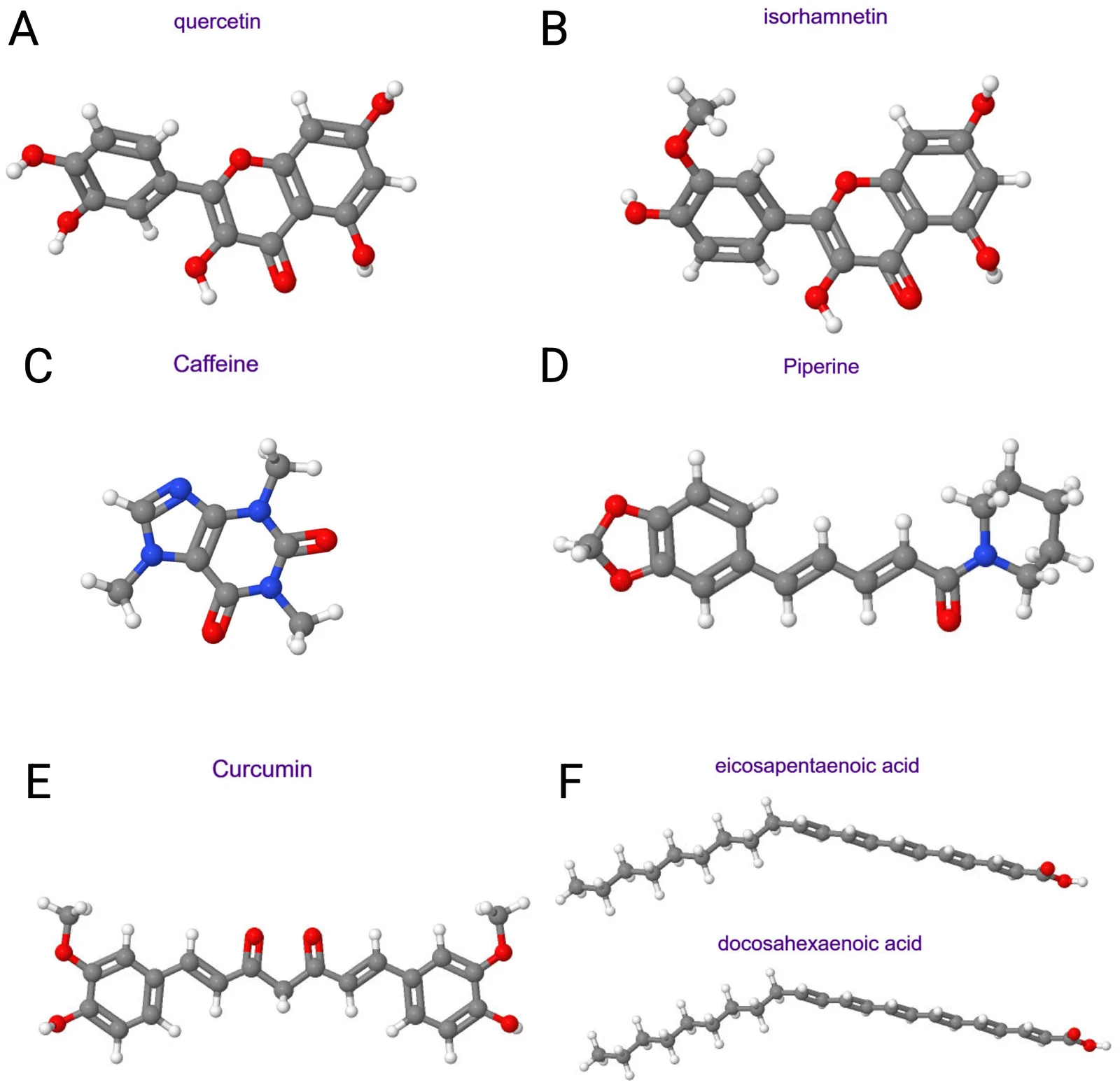
Chemical structure of molecules. 2D structural design of the bioregulatory molecules analyzed in the present review. For their design, we use Biomodel free access website, and Bienfait molecule editor (Bienfait and Ertl, 2013).

The studies in animal models demonstrated that CLO reduced periodontal bone loss, and lower pro-inflammatory mediators like interleukin (IL)-1β, and tumor necrosis factor (TNF)-α (Tanideh et al., 2020) (**Figure 3**). A dose of 90 mg/kg of CLO prevented bone loss and decreased the levels of TNF-α and IL-1β compared to the control group treated with saline solution (Alexandre et al., 2017; Lima et al., 2017). The doses used in these experimental studies are similar to those used in humans, where the dosage is 20 to 40 drops four times a day at a 10% concentration (Silva et al., 2021). Although the dosage is empirical, these concentrations have demonstrated an effective anti-inflammatory effect in homeopathic medicine (Arora et al., 2013; Silva et al., 2021). In this context, the CLO extracts exhibit complex immune-modulating effects, with some studies highlighting lymphocyte activation and anti-inflammatory actions through the reduction of cytokines like IL-1β, IL-6, and TNF-α.

**Figure 3:**
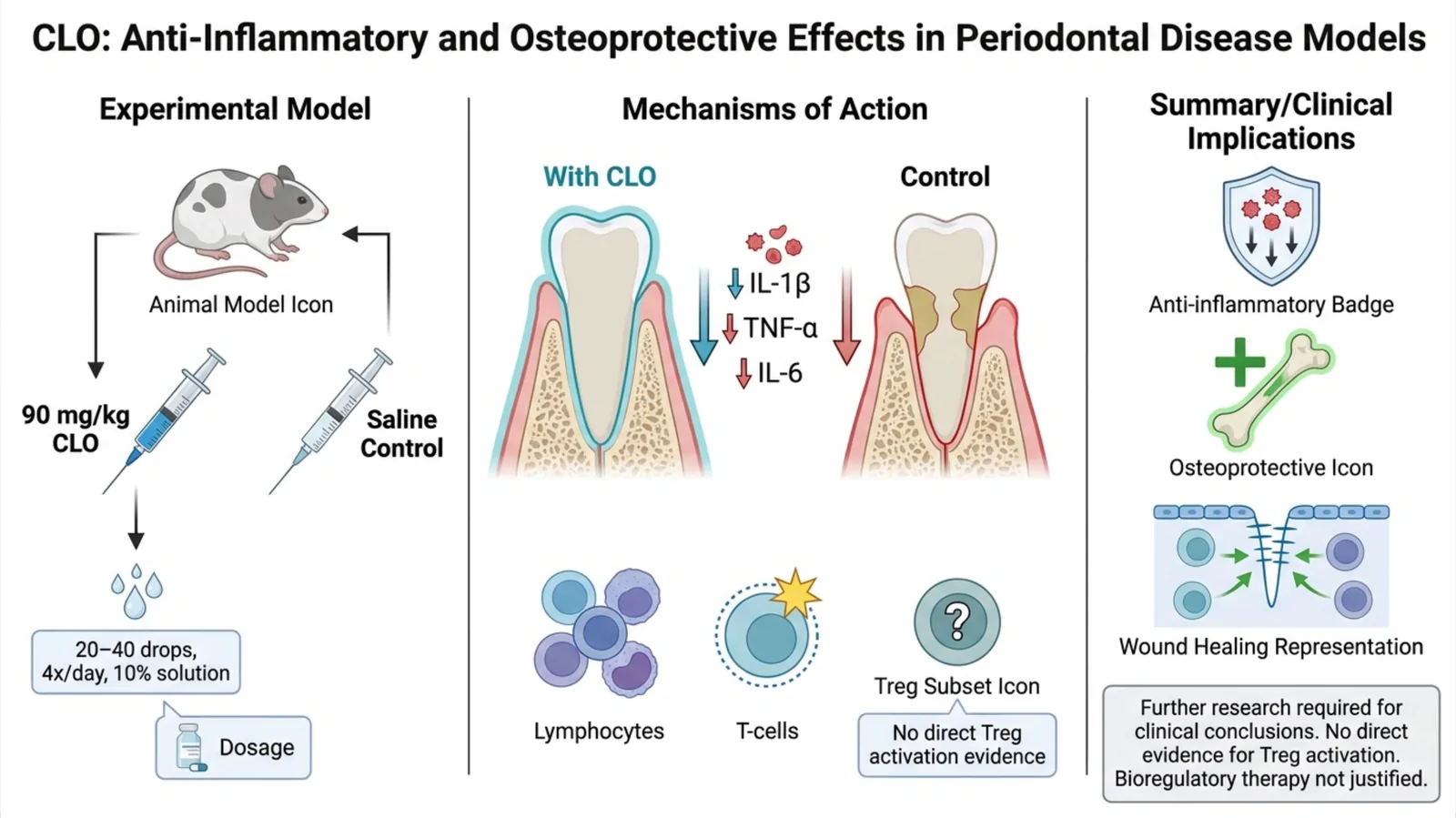
Calendula main findings. Based on the analyzed experimental studies, the image summarize the experimental model methods, the potential mechanisms of action of CLO, and the clinical implications.

Studies show that CLO can activate lymphocytes and influence T-cell responses to aid in wound healing and potentially modulate regulatory T (Treg) subsets (Jiménez-Medina et al., 2006; Golubova et al., 2025). These findings support the anti-inflammatory and osteoprotective potential of CLO in the treatment of periodontitis, although it is not yet possible to draw firm conclusions due to the need for further research and more clinical evidence. Based on the provided search results, there is no direct evidence found regarding CLO’s effect on activating Tregs. Thus, while CLO exhibits a potential as an anti-inflammatory molecule, its use in bioregulatory therapy —a key axis of BrSM— is not yet justified.

### Caffeine

Caffeine is an alkaloid from the methylxanthine group present in coffee and tea. It is a trimethylxanthine alkaloid with the chemical formula C_8_H_10_N_4_O_2_ and with the IUPAC name 1,3,7-trimethyl-3,7-dihydro-1H-purine-2,6-dione (Mody et al., 2025) (**Figure 2C**). The 2D structure is a purine derivative with a fused bicyclic ring system of xanthine (pyrimidine and imidazole) and is distinguished by having three methyl groups at positions N-1, N-3, and N-7 of the xanthine nucleus (Reddy et al., 2024). Caffeine is a colorless, bitter white solid and moderately water-soluble (2g/100 mL at 25 °C). This substance could readily cross the biological membranes of cells and the blood-brain barrier due to its lipophilic properties, which are related to its mechanism of action, primarily as a competitive antagonist of adenosine receptors (A1 and A2A) in the central nervous system (Čižmárová et al., 2025).

Five articles were selected, four of which were studies in humans and one in Wistar rats (Ng et al., 2014; Duarte and Reis, 2015; Han et al., 2016; Struppek et al., 2022; Sari et al., 2023). The evidence was mixed regarding the relationship between coffee consumption and periodontitis. In the experimental study in rats, *Robusta coffee* bean extract demonstrated antibacterial properties against periodontal pathogens and an accelerating effect on alveolar bone repair after administration as a mouthwash at different concentrations (Sari et al., 2023) (**Figure 4**).

**Figure 4:**
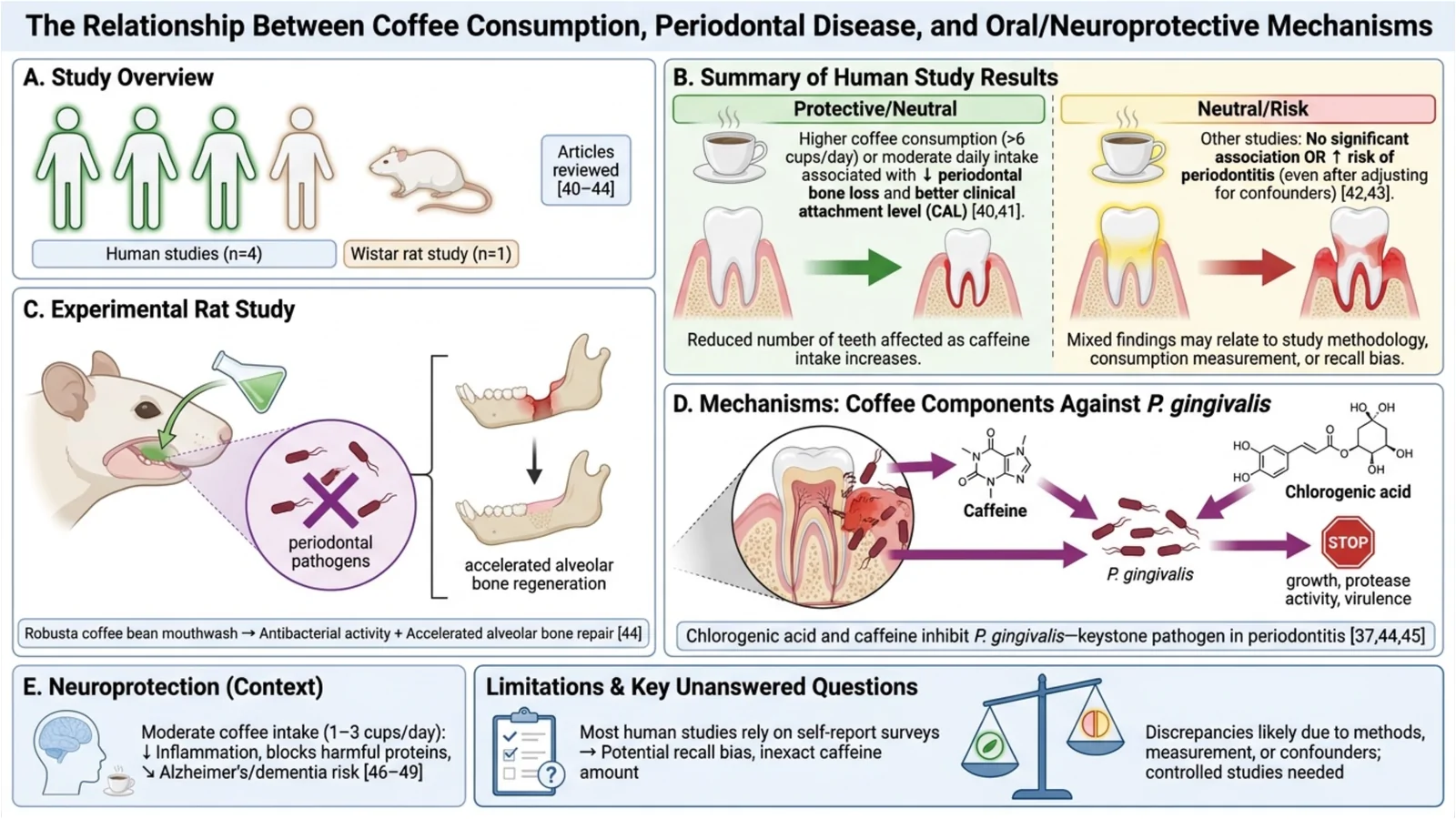
Coffee main findings. Based on both the experimental and human studies analyzed the image represents a summary of the principal methodological aspects of both type of studies, the potential mechanisms of action, and the limitations.

Based on clinical studies, Ng et al. (2014) reported that higher coffee consumption was linked to a small but statistically significant reduction in the number of teeth with periodontal bone loss in adult males, suggesting a protective effect. In another 30-year follow-up study, participants who where higher consumers of coffee/tea (<6 cups/day) or moderate (<1 cup/day) had a better periodontal outcomes —less clinical attachment level loss—. Furthermore, an additional reduction in the number of teeth affected by clinical attachment level (CAL) loss was observed as daily coffee consumption increased (Duarte and Reis, 2015). Conversely, some studies found no significant link between drinking 3–6 cups of coffee daily and the presence or absence of periodontitis, implying neutral or inconclusive results (Han et al., 2016; Struppek et al., 2022).

While most studies show that coffee consumption is a protective factor against developing periodontitis, others indicate that the effect could be neutral or even risky. Regardless, a recent study demonstrated that coffee components, including caffeine and notably chlorogenic acid, possess antimicrobial properties that can inhibit the growth, protease activity, and virulence of *Porphyromonas gingivalis* —the keystone pathogen directly link with disbiosis associated with periodontitis onset (Tsou et al., 2019; Sari et al., 2023; Mody et al., 2025). Additionally, current research strongly links moderate coffee consumption to neuroprotection, significantly lowering the risk of dementia and Alzheimer’s disease by reducing inflammation and blocking harmful proteins, with benefits seen around 1–3 cups daily (Carman et al., 2014; Wasim et al., 2020; Yelanchezian et al., 2022; Wang et al., 2024).

Otherwise, while some human clinical studies reported a possible association between high coffee consumption and reduced periodontal bone loss (Ng et al., 2014; Duarte and Reis, 2015), other studies observed an inverse relationship, where high coffee consumption was associated with a higher risk of periodontitis, even after adjusting for confounding factors such as age, smoking, and co-morbidities (Han et al., 2016; Struppek et al., 2022). The only experimental study conducted in rats, reported positive effects of *Robusta coffee* bean extract, with antibacterial activity and accelerated alveolar bone repair when administered as a mouthwash (Sari et al., 2023). This discrepancy between the findings could be due to methodological differences, the type and amount of consumption assessed, or the specific source of caffeine considered. It is noteworthy to note that, in clinical studies, coffee consumption was assessed using validated self-report questionnaires, which can introduce participant recall bias and lead to inaccurate consumption estimates. Furthermore, this method does not allow for the control of variables such as the actual concentration of caffeine ingested; therefore, controlled studies are needed to define its role in the development or prevention of periodontal disease. This is particularly relevant considering that coffee is one of the most consumed beverages worldwide (Struppek et al., 2022).

### Piperine

Piperine is the principal alkaloid of black pepper *(Piper nigrum)*, responsible for its pungency. Its chemical formula is C_17_H_19_NO_3_ with IUPAC name (2E,4E)-5-(2H-1,3-Benzodioxol-5-yl)-1-(piperidin-1-yl)penta-2,4-dien-1-one (Frolov and Vereshchagin, 2023; Shahane et al., 2023). The structure features comprise a methylenedioxyphenyl ring connected to a conjugated aliphatic chain (diene) that is linked to a piperidine ring via a carbonyl amide bond (**Figure 2D**). Piperine is a pale-yellow crystalline solid with very low water solubility (logP ≈ 3.1), and it is highly soluble in organic solvents, particularly chloroform, ethanol, acetone, and ethyl acetate (Meghwal and Goswami, 2013; Ezawa et al., 2021). This substance is known as a biopotentiator that works by inhibiting enzymes that break down drugs, such as CYP3A4 and P-glycoprotein. As a result, it increases the bioavailability of other compounds, like curcumin. In addition, it modulates signaling pathways, including STAT-3 and NK-κB to produce its effects (Mitra et al., 2021).

Two experimental studies related to piperine were selected, in which periodontitis was induced by ligature in Wistar and Holtzman rats, and piperine administered via oral gavage in different doses (Dong et al., 2015; Guimaraes-Stabili et al., 2019). The evidence highlights the potential of piperine as a therapeutic agent in the management of periodontitis (**Figure 5**). According to Dong et al. (2015), piperine inhibited alveolar bone loss and improved trabecular microstructure in a dose-dependent manner. Furthermore, it significantly reduced inflammatory infiltration into soft tissues and limited the areas of collagen fiber degradation at all doses evaluated. At the molecular level, piperine at a dose of 100 mg/kg significantly decreased the expression of IL-1β, MMP-8, and MMP-13 —key mediators in inflammatory processes and periodontal tissue destruction—. Otherwise, Guimaraes-Stabili et al. (2019) demonstrated that both curcumin and piperine promoted a positive effect on tissue repair when administered as a single dose. In animals treated with piperine, an increase in IL-10 was detected, together with a reduced progression of periodontitis and inflammation.

**Figure 5:**
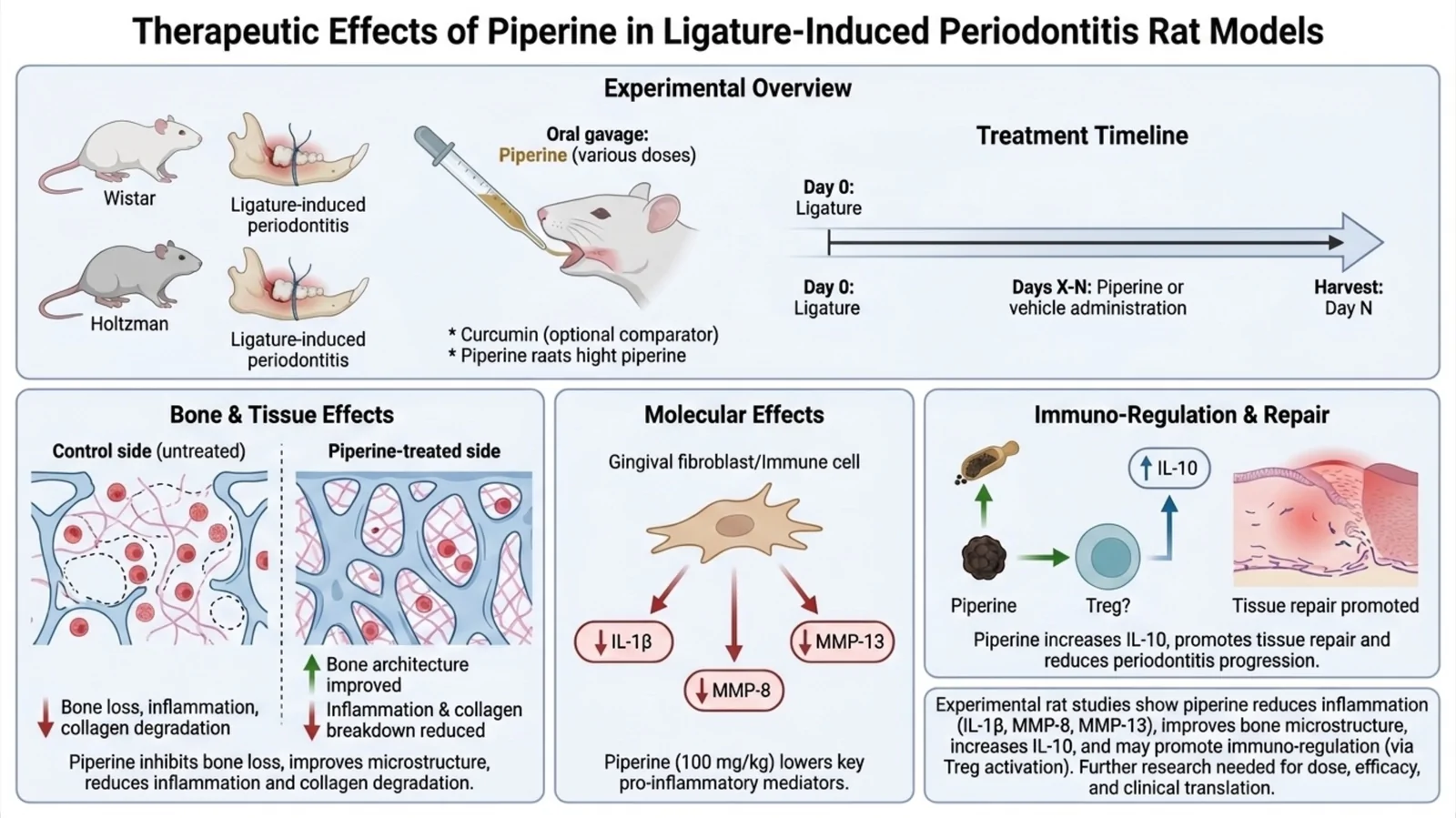
Piperine main findings. Based on the experimental studies the image represents a summary of the methods, and the effects at tissular, molecular, and immunological levels.

Although the evidence is again insufficient to draw definitive conclusions, the results suggest that it could reduce pro-inflammatory mediators (IL-1β, MMP-8, and MMP-13), improve bone architecture, and increase the presence of IL-10 in periodontal tissues, suggesting an immune-regulatory effect, at least, in the experimental models (Dong et al., 2015; Guimaraes-Stabili et al., 2019). This indicates that it could represent a promising line of research as a potential inducer of bioregulation via Treg activation; however, further research is required to clearly determine its therapeutic dose, efficacy, and applicability.

### Curcumin

Curcumin is the main polyphenol of the *Curcuma longa* rhizome. Its structure is notable for its symmetrical and tautomeric capacity (Priyadarsini, 2014). Its chemical formula is C_21_H_20_O_6_ and, structurally, it is a linear, symmetrical molecule consisting of two 4-hydroxy-3-methoxyphenyl rings connected by a heptadienedione chain (**Figure 2E**). The IUPAC name is (1E,6E)-1,7-Bis(4-hydroxy-3-methoxyphenyl)hepta-1,6-diene-3,5-dione. The 2D structure shows ortho-methoxy phenolic rings linked by a seven-carbon chain containing a bis-α, β-unsaturated β-diketone group, and the 3D conformation is relatively planar, allowing cell membrane penetration. Curcumin is hydrophobic (logP ≈ 3.3) and poorly soluble in water. Moreover, it is soluble in ethanol, DMOS, and acetone. It exhibits keto-enol tautomerism in neutral or acidic solutions, where the keto form predominates, while in the solid state and alkaline solutions, it is more stable in the enol form and absorbs UV-visible light (≈ 420 nm) (Zhai et al., 2020; Urošević et al., 2022; Ciuca and Racovita, 2023). The mechanism of action related to its structure includes acting as a potent antioxidant and anti-inflammatory agent, and inhibiting inflammatory mediators such as nuclear factor k-B (NF-kB), COX-2, and LOX. Also, its structure allows it to act as a free radical scavenger and metal chelator (Sharifi-Rad et al., 2020; Toraya et al., 2020).

Twenty-three studies on curcumin were selected: 10 clinical trials (Bhatia et al., 2014; Anitha et al., 2015; Anuradha et al., 2015; Nagasri et al., 2015; Elavarasu et al., 2016; Hugar et al., 2016; Nasra et al., 2017; Ravishankar et al., 2017; Rahalkar et al., 2021; Abdel-Fatah et al., 2023), 1 comparative study (Mohammad, 2020), and 12 experimental studies in rats (Hosadurga et al., 2014; Bakır et al., 2016; Akpinar et al., 2017; Corrêa et al., 2017; Xiao et al., 2018; Pimentel et al., 2020; Iova et al., 2021; Sha et al., 2021; Mohammad et al., 2022; Mohammad et al., 2023; Wang et al., 2023; Rani Safitri et al., 2024). Animal model studies demonstrate that curcumin serves as an effective adjunct in treating periodontitis by modulating inflammatory response, preserving periodontal tissue, and reducing disease-related clinical/biochemical parameters (Hosadurga et al., 2014; Bakır et al., 2016; Akpinar et al., 2017; Corrêa et al., 2017; Xiao et al., 2018; Pimentel et al., 2020; Iova et al., 2021; Sha et al., 2021; Mohammad et al., 2022; Mohammad et al., 2023; Wang et al., 2023; Rani Safitri et al., 2024) (**Figure 6**). Also, the studies confirm positive results in reducing periodontal inflammation, preserving tissue, and improving clinical/biochemical markers. In fact, the reduction in alveolar bone loss effect was achieved with doses of 100 mg/kg/day for 30 days (Corrêa et al., 2017) and doses of 75 and 150 mg/kg/day for 11 days (Akpinar et al., 2017). In the context of diabetes-related co-morbidities the combination of curcumin with insulin prevents periodontal deterioration by reducing alveolar bone loss, suppressing inflammatory markers, and combating oxidative stress (Pimentel et al., 2020; Iova et al., 2021). On the other hand, Wang et al. (2023) proposed that curcumin exerts its bone-protective effect by inhibiting ferroptosis, a type of Iron-dependent, non-apoptotic death, specifically by reducing oxidative stress and lipid peroxidation in bone cells. Thus, curcumin mitigates bone loss by suppressing reactive oxygen species (ROS) production and increasing enzymes like GPX4, crucial for mitigating ferroptosis-induced damage.

**Figure 6:**
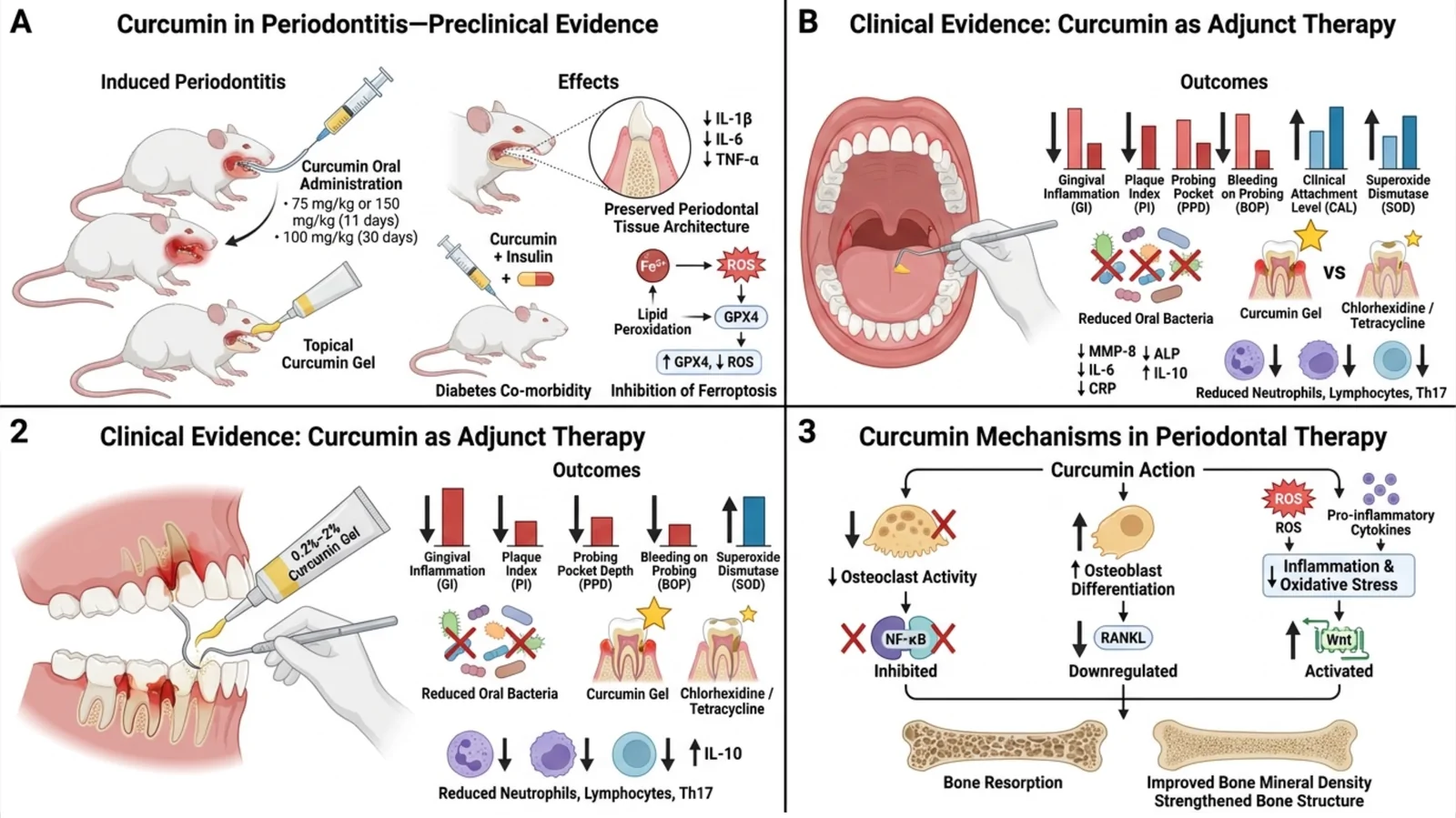
Curcumin main findings. Based on both the experimental and human studies the image represents a summary of the experimental methods, the clinical evidence as adjunct therapy, and the potential mechanisms of the curcumin in periodontal therapy.

The clinical trials reviewed evaluated the efficacy of curcumin as an adjunct to non-surgical periodontal therapy in patients with periodontitis. Curcumin was applied topically in gels with concentrations ranging from 0.2% to 2%. Overall, the studies reported a significant reduction in clinical parameters, curcumin gel was more effective than oral rehydration therapy in inhibiting the growth of oral bacteria (Bhatia et al., 2014), and a significant decrease in the concentration of pro-inflammatory mediators (Mohammad, 2020). Also, local application of 2% curcumin gel acts as a potent, biocompatible adjunct to scaling and root planing, significantly reducing gingival inflammation, plaque index, and probing pocket depth (Hosadurga et al., 2014; Xiao et al., 2018; Abdel-Fatah et al., 2023). Furthermore, following successful periodontal therapy, patients often experience reduced inflammation-mediated destruction of soft/hard tissues and inhibited osteoclastogenesis, together with improvements in bleeding on probing, probing pocket depth, and clinical attachment level, with sustained clinical gain during follow-up (Anuradha et al., 2015; Nagasri et al., 2015; Nasra et al., 2017; Ravishankar et al., 2017; Rahalkar et al., 2021; Sha et al., 2021). Besides, Curcumin treatment significantly increases superoxide dismutase levels in the subgingival environment, confirming its role as a potent antioxidant in managing periodontitis (Elavarasu et al., 2016). Finally, the curcumin gel, used as an adjunct to scaling and root planing, is more effective than chlorhexidine gel in reducing probing depth and improving the clinical attachment level in patients with periodontitis, with studies showing superior reduction in periodontal pocket indices (Anitha et al., 2015; Hugar et al., 2016).

Mohammad et al. (Mohammad et al., 2022; Mohammad et al., 2023) reported that local administration of curcumin as an adjunct to non-surgical periodontal therapy (root scaling and planing) demonstrates significant anti-inflammatory effects, comparable to or exceeding standard treatments like tetracycline. Studies show it reduces key inflammatory biomarkers including MMP-8, IL-6, CRP, and alkaline phosphatase, while increasing anti-inflammatory IL-10 levels. Furthermore, curcumin reduces neutrophils and lymphocytes and lowers local Th17 cell count and IL-17 production, suggesting a targeted, local action in periodontal pockets (Bakır et al., 2016; Rani Safitri et al., 2024).

Considerable evidence and various types of studies with promising results were found. In preclinical studies, curcumin was administered both via nasogastric tube and through topical application, and in both cases were observed a significant decrease in alveolar bone loss, modulation of pro-inflammatory cytokines (IL-1β, IL-6, TNF-α), and an improvement in periodontal tissue architecture (Corrêa et al., 2017; Sha et al., 2021; Rani Safitri et al., 2024). In clinical trials, topical administration was preferred, and a significant improvement in clinical parameters such as periodontal index, gingival index, probing pocket depth, and clinical attachment level was demonstrated (Bhatia et al., 2014; Hosadurga et al., 2014; Anitha et al., 2015). This is likely due to the low bioavailability of curcumin when administered orally; it is poorly absorbed, and only trace amounts of the compound appear in the blood. This would justify its direct application to maximize contact time with the mucosa, improve treatment adherence, and enhance its therapeutic effect (Anuradha et al., 2015). Furthermore, in animal studies, systemic doses that yielded positive results ranged from 75 mg/kg to 150 mg/kg, which would represent a high dose and impractical administration in humans.

Taken together, the scientific research strongly suggests curcumin helps reduce bone destruction by inhibiting osteoclast activity, promoting osteoblast differentiation, and reducing inflammation and oxidative stress. Curcumin achieves this by targeting key pathways like NF-kB, receptor of activator NF-κB ligand (RANKL), and *Wnt*, improving bone mineral density and strengthening bone structure (Peddada et al., 2015; Hatefi et al., 2018; Ke et al., 2024; Wang, 2024; Mohammad et al., 2025).

### ω-3 fatty acids

The polyunsaturated fatty acids like eicosapentaenoic acid (EPA) with formula C_20_H_30_O_2_ with IUPAC name (5Z,8Z,11Z,14Z,17Z)-icosa-5,8,11,14,17-pentaenoic acid; and the docosahexaenoic acid (DHA) with formula C_22_H_32_O_2_ and IUPAC name is (4Z,7Z,10Z,13Z,16Z,19Z)-docosa-4,7,10,13,16,19-hexa-enoic-acid; whose chemical formula varies depending on the specific acid, share a long-chain hydrocarbon structure of the fatty acids with 5–6 *cis* double bonds and a terminal carboxyl group chol (Cholewski et al., 2018; Shahidi and Ambigaipalan, 2018) (**Figure 2F**). The term ω-3 indicates that the first double bond is located at the third carbon counting from the methyl end of the chain. They are considered as liquid oils and highly lipophilic (logP ≈ 7-9), and this allows them to be integrated and incorporated readily into the cell membranes (Pazderka et al., 2020).

Twenty studies related to ω-3 were selected, of which 4 correspond to experimental studies in animals (Jalal et al., 2020; Ozaki et al., 2020; Doğan and Doğan, 2021; González-Alva et al., 2024) and 16 to studies in humans (Murali et al; Deore et al., 2014; Naqvi et al., 2014; Jauhiainen et al., 2016; Keskiner et al., 2017; Umrania et al., 2017; Rampally et al., 2019; Kujur et al., 2020; Stańdo et al., 2020; Maybodi et al., 2022; Mewes et al., 2022; Stańdo-Retecka et al., 2023; Farahmand et al., 2024; Li et al., 2024; Prasanth et al., 2024; Savran and Sağlam, 2024), mainly clinical trials (**Figure 7**).

**Figure 7:**
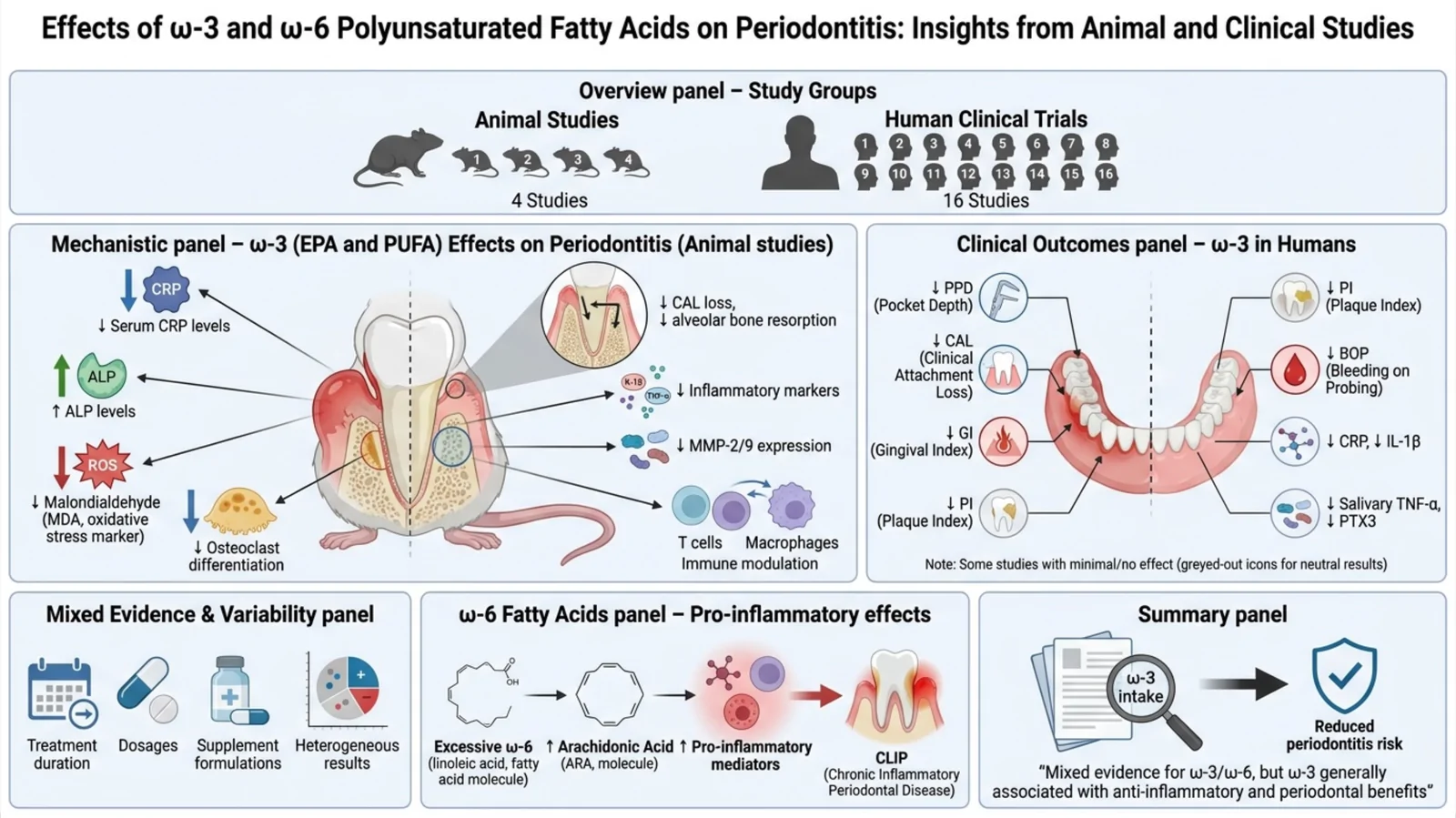
ω-3 and ω-6 main findings. Based on both the experimental and human studies analyzed the image represents a summary of the mechanistic effects of poly-unsaturated fatty acids ω-3, and ω-6 on both animals and humans. Also, the image shows that the evidence has mixed and variable quality, and propose the potential pro-inflammatory effects.

Experimental studies in rats with periodontitis demonstrate that EPA significantly reduces serum C-reactive protein (CRP) levels to near-healthy values within 7 days (Jalal et al., 2020). Additionally, EPA treatment boosts ALP levels and decreases malondialdehyde —an indicator of oxidative stress, while inhibiting osteoclast differentiation to mitigate bone reduction and tissue damage (Ozaki et al., 2020). Dogan et al. demonstrate that dietary supplementation with ω-3 polyunsaturated fatty acids (PUFA) significantly reduces clinical attachment level loss, decrease alveolar bone resorption, and lower inflammatory markers in rats with induced periodontitis (Doğan and Doğan, 2021). Prolonged PUFA supplementation mitigates periodontitis in murine models by reducing bone destruction and inflammation. This effect is achieved through the down-regulation of MMP-2 and MMP-9 and by modulating the immune-response, including lowering pro-inflammatory cytokines (González-Alva et al., 2024). Deore et al. (2014) and Naqvi et al. (2014) observed improvements in clinical parameters, as well as in CRP and IL-1β levels in gingival crevicular fluid, although without changes in serum pro-inflammatory markers. Other studies also showed significant reductions in probing pocket depth and clinical attachment level, and short- and medium-term anti-inflammatory effects (Murali et al; Kujur et al., 2020; Stańdo et al., 2020; Maybodi et al., 2022; Stańdo-Retecka et al., 2023; Prasanth et al., 2024; Savran and Sağlam, 2024), in addition to a significant reduction in gingival index, plaque index, and bleeding on probing (Farahmand et al., 2024). Regarding pro-inflammatory biomarkers, the studies observed a significant reduction in salivary TNF-α (Keskiner et al., 2017), and lower levels of Pentraxin 3 (PTX3) —a protein belonging to the CRP family that has proven to be a highly sensitive and specific marker in inflammatory states— (Rampally et al., 2019). Evidence on the periodontal benefits of ω-3 and ω-6 fatty acids is mixed, with some studies showing minimal impact. While some research suggests no association between ω-3/ω-6 intake and periodontal health in certain populations, other studies indicate only a minor decrease in bleeding on probing, or no significant, long-term clinical differences compared to control (Jauhiainen et al., 2016; Umrania et al., 2017; Mewes et al., 2022; Li et al., 2024).

Regarding the results the ω-3/ω-6 administration showed a significant reduction in inflammatory biomarkers such as CRP (Jalal et al., 2020), a decrease in oxidative stress and bone resorption, stimulation of osteoblastic activity (Ozaki et al., 2020; Doğan and Doğan, 2021), improvement in clinical parameters such as probing pocket depth, cinical attachment level, and gingival index (Murali et al; Naqvi et al., 2014; Kujur et al., 2020). However, the results have been heterogeneous, and some studies did not report significant benefits or found improvements only in certain clinical parameters or biomarkers (Umrania et al., 2017; Rampally et al., 2019). This variability could be due to differences in treatment duration, dosages used, and supplement formulation. However, recent observational studies suggest a possible inverse relationship between ω-3 intake and the risk of periodontitis (Li et al., 2024). It is noteworthy to mention that excessive intake of ω-6 polyunsaturated fatty acids —particularly linoleic acid— can increase arachidonic acid (ARA) production, leading to higher levels of pro-inflammatory mediators, contributing to a CLIP (Patterson et al., 2012; Innes and Calder, 2018; DiNicolantonio and O'Keefe, 2021).

### Heterogeneity of studies

The studies analyzed in this qualitative systematic review were characterized by being heterogeneous; therefore, no statistical analysis of the data was performed. Among the experimental animal studies, differences were observed in the species used, age, and experimental time. While most studies were conducted on Wistar rats, other species were also used, such as Holtzman rats (Guimaraes-Stabili et al., 2019), Sprague-Dawley rats (Tanideh et al., 2020), and C57/BL mice (Ozaki et al., 2020; Wang et al., 2023; González-Alva et al., 2024). Regarding the time of exposure to the intervention, it varied considerably, from 6 days (Hosadurga et al., 2014), 30 days (Corrêa et al., 2017; Pimentel et al., 2020), 44 days (Doğan and Doğan, 2021), to 70 days (González-Alva et al., 2024). In the human studies, there were differences in study design, participant age, sex ratio, and duration of intervention administration. The variability in the criteria used to define periodontitis makes it difficult to standardize case definitions. While most use the gingival index and probing pocket depth, several studies link the diagnosis of periodontitis to certain probing depths. In other cases, the analysis is more population-based, using the community periodontal index treatment needs, and in still others, patients already diagnosed by other professionals are used, with unspecified criteria.

### Analysis of the risk of bias in the studies

The RoB in the studies included in this systematic review was assessed using three tools, as appropriate. For observational studies, the NOS was applied: 9 studies were analyzed and classified as high (Ng et al., 2014; Duarte and Reis, 2015; Jauhiainen et al., 2016; Mewes et al., 2022; Li et al., 2024), or moderate (Han et al., 2016; Mohammad, 2020; Struppek et al., 2022; Savran and Sağlam, 2024) quality based on their scores (**Supplementary Figure 1**). In general, the selection and comparability domains showed good results, with scores close to the maximum in most cases. However, the exposure domain yielded lower scores, primarily for the item related to the non-response rate, because some studies did not report this information, preventing them from being scored on this criterion (Duarte and Reis, 2015; Han et al., 2016; Mohammad, 2020; Mewes et al., 2022; Struppek et al., 2022; Savran and Sağlam, 2024). Despite this, all the evaluated studies obtained overall scores between 6 and 9 stars, indicating moderate to high methodological quality and meeting the criteria necessary to support the findings of this review. If we specify the studies analyzed using this tool, we can indicate that those that obtain a high quality of evidence are the caffeine (Ng et al., 2014; Duarte and Reis, 2015), and ω-3 (Jauhiainen et al., 2016; Mewes et al., 2022; Li et al., 2024) studies. In addition, those rated as moderate were those performed on caffeine (Han et al., 2016; Struppek et al., 2022), curcumin (Mohammad, 2020), and ω-3 (Savran and Sağlam, 2024). This means that 80% of the studies on caffeine are observational with high (40%) or moderate (40%) quality, 4% of the studies on curcumin are observational and of moderate quality, and 19% of the studies on ω-3 are also observational with high (16%) or moderate (3%) quality. The most important aspect of these studies is that they allow us to establish, with some certainty, that caffeine consumption is associated with less bone loss and that the use of ω-3 fatty acids were negatively associated with the risk of developing periodontitis.

To assess the RoB in clinical trials, the RoB2 tool was used (**Supplementary Figure 2**). Of the 22 studies analyzed, 5 were assessed as “low risk” (Deore et al., 2014; Anitha et al., 2015; Anuradha et al., 2015; Maybodi et al., 2022; Prasanth et al., 2024), 14 as having “some concerns” (Murali et al; Bhatia et al., 2014; Naqvi et al., 2014; Nagasri et al., 2015; Hugar et al., 2016; Keskiner et al., 2017; Umrania et al., 2017; Rampally et al., 2019; Kujur et al., 2020; Stańdo et al., 2020; Rahalkar et al., 2021; Abdel-Fatah et al., 2023; Stańdo-Retecka et al., 2023), and 3 as having “high risk” (Elavarasu et al., 2016; Nasra et al., 2017; Ravishankar et al., 2017). The domains where concerns were most frequently concentrated were domain 2 (bias due to deviations from planned interventions) and domain 4 (bias in outcome measurement). In domain 2, concerns arose mainly from the lack of adequate measures to ensure that participants and caregivers were blinded to the intervention they were receiving, as well as from the absence of information on possible deviations and their impact on the study results. In domain 4, most studies did not provide clear information on whether outcome assessors were blinded to the assigned intervention, which could have influenced the objectivity of the measurement. These methodological limitations prevented a more favorable classification regarding the RoB. Studies that received an overall “high risk” rating did so because they presented “some concerns” in four or five of the evaluated domains, reflecting an accumulation of methodological deficiencies that could affect the reliability of their results. Regarding high-quality methodological clinical trials, studies on curcumin (Anitha et al., 2015; Anuradha et al., 2015), ω-3 (Deore et al., 2014; Maybodi et al., 2022; Prasanth et al., 2024) stand out, which is equivalent to 9% and 15% of the studies analyzed, respectively. About the clinical trials conducted on curcumin (Bhatia et al., 2014; Nagasri et al., 2015; Hugar et al., 2016; Rahalkar et al., 2021; Abdel-Fatah et al., 2023), ω-3 (Murali et al; Naqvi et al., 2014; Keskiner et al., 2017; Umrania et al., 2017; Rampally et al., 2019; Kujur et al., 2020; Stańdo et al., 2020; Stańdo-Retecka et al., 2023), they were considered to be of moderate quality, which represent the 22% and 40%. Finally, the studies conducted in curcumin (Elavarasu et al., 2016; Nasra et al., 2017; Ravishankar et al., 2017) were categorized as high risk and should not be considered for any clinical decision due to the methodological bias. These analyses inform us that, for the most part, the results from clinical trials conducted on curcumin and ω-3 should be considered of good quality and, therefore, replicable in future studies or therapies. Of these, the best evidence is that produced in ω-3 fatty acids.

For animal models, the RoB was assessed using the SYRCLE tool (**Supplementary Figure 3**). In domain 1, which corresponds to random sequence generation, 63.6% of the studies were classified as “Yes” (Hosadurga et al., 2014; Dong et al., 2015; Akpinar et al., 2017; Corrêa et al., 2017; Xiao et al., 2018; Jalal et al., 2020; Ozaki et al., 2020; Tanideh et al., 2020; Doğan and Doğan, 2021; Sha et al., 2021; Mohammad et al., 2023; Wang et al., 2023; González-Alva et al., 2024; Rani Safitri et al., 2024); however, 9 of them indicated that randomization was performed without detailing the procedure, which was classified as “Yes”. Other domains with good results were domain 7 (50.0% “Yes”), which refers to blinding of the evaluator of the results, and domain 10 (40.9% “Yes”), which reports other possible sources of bias. Conversely, domain 6 was the most deficient, with 45.5% of studies marked “No”, indicating that nearly half of the studies lacked blinding when analyzing the results (Hosadurga et al., 2014; Jalal et al., 2020; Pimentel et al., 2020; Tanideh et al., 2020; Doğan and Doğan, 2021; Iova et al., 2021; Sha et al., 2021; Mohammad et al., 2022; Mohammad et al., 2023; Rani Safitri et al., 2024). Domain 3 followed, with 36.4% “No,” due to a lack of information on whether adequate allocation concealment was performed. The remaining domains showed a high proportion of “Unclear” results: domain 8 had 81.8%, domains 2 and 4 had 77.3%, domain 5 had 72.7%, and domain 9 had 59.1%, indicating that the information about the methodology was not sufficient or clear to make a judgment about the RoB. With regard to these studies, none stand out for methodological rigor, leaving this evidence with a low to moderate quality. Among the studies evaluated with this tool are those carried out in CLO (Alexandre et al., 2017; Lima et al., 2017; Tanideh et al., 2020), caffeine (Sari et al., 2023), piperine (Dong et al., 2015; Guimaraes-Stabili et al., 2019), curcumin (Hosadurga et al., 2014; Bakır et al., 2016; Akpinar et al., 2017; Corrêa et al., 2017; Xiao et al., 2018; Pimentel et al., 2020; Iova et al., 2021; Sha et al., 2021; Mohammad et al., 2022; Mohammad et al., 2023; Wang et al., 2023; Rani Safitri et al., 2024), ω-3 (Jalal et al., 2020; Ozaki et al., 2020; Doğan and Doğan, 2021; González-Alva et al., 2024). Overall, the main deficiencies in the methodology of the evaluated studies were related to the lack of information provided and the absence of blinding strategies during the different stages of the development of the studies. It is noteworthy to note that the 100% of the studies conducted in CLO and piperine are experimental with moderate quality, and the 52%, 20%, and 20% of the studies in curcumin, caffeine, and ω-3 fatty acids respectively were experimental with moderate quality. While experimental studies do not allow us to establish clinical practices, they are important for laying the molecular basis of the physiological effect of molecules on pathological processes.

In general terms, the best evidence comes from studies on curcumin and ω-3 fatty acids followed by caffeine, leaving CLO, curcumin, and piperine as potentially beneficial molecules. However, experimental studies need to increase in both quality and quantity to move toward preclinical or clinical trials and consider CLO, curcumin, and piperine as adjuvant therapeutic molecules.

## Bioregulatory periodontal medicine

The different natural molecules used as adjuvants in the treatment of periodontitis showed therapeutic potential and mechanisms that align coherently with the fundamental principles of BrSM. Compounds such as curcumin, ω-3 fatty acids, and caffeine have been shown to act through multiple signaling pathways, exerting antimicrobial, anti-inflammatory, antioxidant, immuno-modulatory, and osteoprotective effects, which contribute to restoring tissue homeodynamics without aggressively blocking the body’s physiological responses. This type of regulatory action on multiple therapeutic targets is what is sought in BrSM, since it allows addressing not only the symptoms of the disease, but also the underlying pathophysiological mechanisms, respecting the complexity of the biological system and promoting its functional recovery (Goldman et al., 2015). Furthermore, given their natural origin and minimal adverse effects, according to current evidence, these molecules are viable therapeutic options within the BrSM model, which prioritizes safety, biocompatibility, and early intervention before disease onset. Their use as an adjunct in periodontitis management could represent a step toward more personalized, less invasive, and more sustainable long-term treatments. Otherwise, the molecules as CLO, curcumin and piperine could act in a similar manner, but the quality of studies and their bias indicate us that this evidence must be considered them with caution. It is essential to advance high-quality clinical research that allows validating its efficacy, safety, and therapeutic applicability in humans, under rigorous methodological criteria and with a truly integrative approach, as proposed by the BrSM. The natural molecules selected in this study exhibit different mechanisms of action through which they produce therapeutic effects in the context of periodontitis. These compounds act by modulating multiple pathophysiological pathways, such as inflammation, oxidative stress, antimicrobial activity, and the immune response (**Figure 8**).

**Figure 8:**
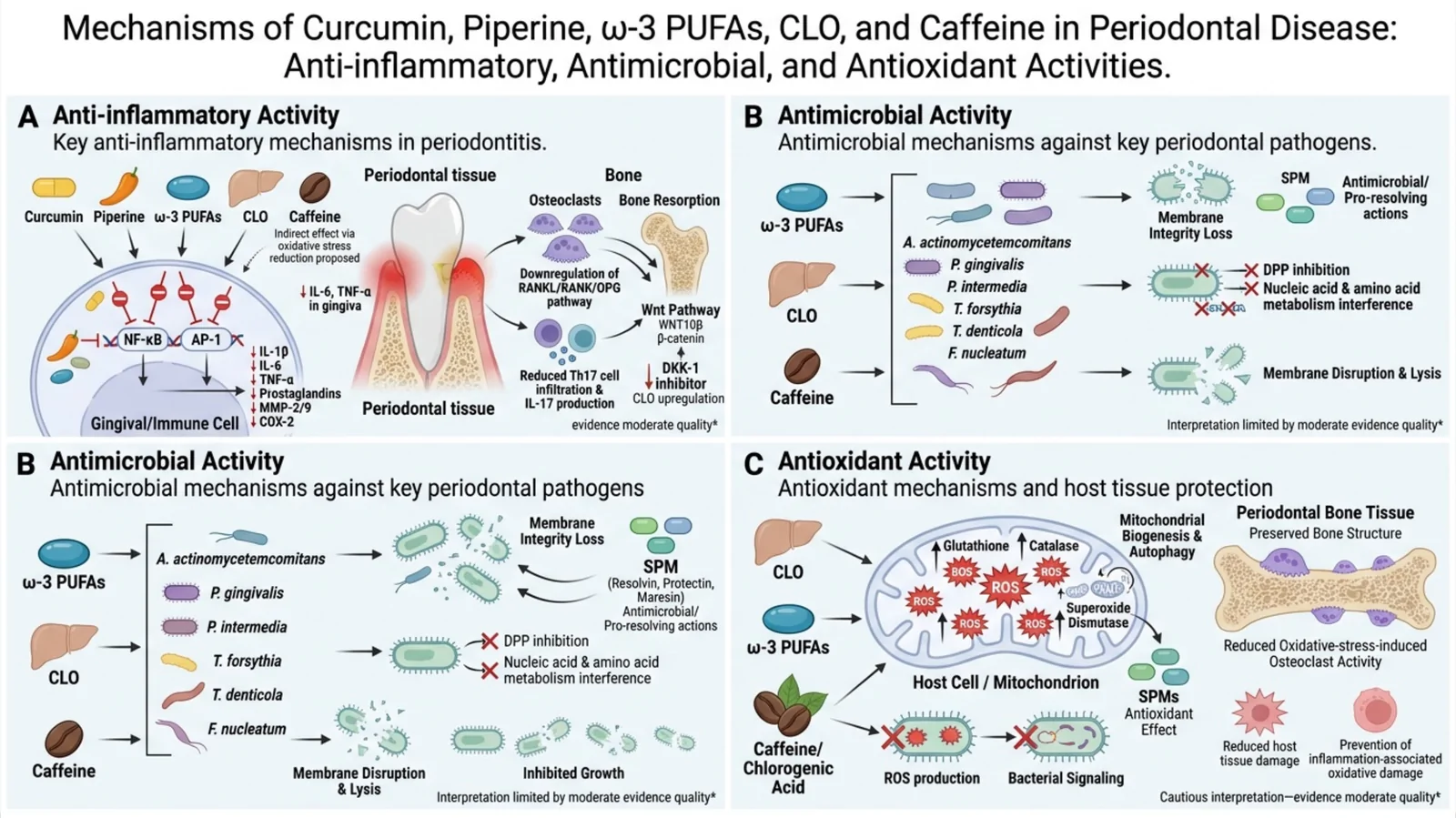
Anti-inflammatory, antimicrobial, and antioxidant activities of the bioregulatory molecules. The image represents the summary of the main effects of the bioregulatory molecules in the periodontal tissues. **(A)** Shows the anti-inflammatory activity of curcumin, piperine, ω-3, CLO, and caffeine at nuclear, and cellular level. **(B)** Antimicrobial activity of ω-3, CLO, and caffeine, and **(C)** The anti-oxidant activity of CLO, ω-3, and caffeine at mitochondrial level. All the evidence represented in the image must be interpreted with caution due to the moderate quality of the evidence.

### Anti-inflammatory activity

The anti-inflammatory activity of curcumin, piperine, and ω-3 PUFAs has been demonstrated, primarily attributed to its ability to modulate intracellular signaling pathways and transcription factors, such as NF-κB and activator protein-1 (AP-1). These molecules are responsible for activating the expression of genes related to inflammation. By inhibiting these pathways, curcumin suppresses the production of IL-1β, IL-6, and TNF-α, prostaglandins, MMP-2 and MMP-9, and COX-2 (Hosadurga et al., 2014; Dong et al., 2015; Alexandre et al., 2017; Lima et al., 2017; Tanideh et al., 2020; González-Alva et al., 2024; Savran and Sağlam, 2024). In periodontitis models, a decrease in IL-6 and TNF-α has been observed in gingival tissue, along with a down-regulation of the RANKL/RANK/OPG pathway, which is related to bone resorption (Corrêa et al., 2017). Furthermore, it reduces Th17 cell infiltration and IL-17 production (Bakır et al., 2016), and in gingival fibroblasts inhibits LPS-induced NF-κB activation, and decrease IL-1β and TNF-α expression (Xiao et al., 2018). This evidence should be analyzed with caution since the studies that support this evidence had a moderate quality rating according to the bias tools used (Hosadurga et al., 2014; Dong et al., 2015; Bakır et al., 2016; Alexandre et al., 2017; Corrêa et al., 2017; Lima et al., 2017; Xiao et al., 2018; Tanideh et al., 2020; González-Alva et al., 2024; Savran and Sağlam, 2024).

Furthermore, it has been reported that CLO upregulate the *Wnt* pathway through increased WNT10β and β-catenin levels and reduced DKK-1 —a WNT pathway inhibitory protein—, which may be linked to its anti-inflammatory action in bone tissue (Lima et al., 2017). In the case of caffeine, there are no conclusive studies on its direct anti-inflammatory effect in periodontitis; however, it has been proposed that its ability to reduce oxidative stress could indirectly modulate inflammation (Sari et al., 2023). Further studies are needed to confirm this mechanism in periodontal tissues because the method quality of both studies is moderate (Lima et al., 2017; Sari et al., 2023).

### Antimicrobial activity

Several studies have demonstrated that ω-3 PUFAs possess antimicrobial effects against periodontal pathogens. They have been reported to directly inhibit the growth of *Aggregatibacter actinomycetemcomitans*, *Porphyromonas gingivalis*, *Prevotella intermedia*, *Tannerella forsythia*, *Treponema denticola*, and *Fusobacterium nucleatum* (González-Alva et al., 2024; Savran and Sağlam, 2024). Among the possible mechanisms involved are the alteration of bacterial membrane integrity by PUFAs and the production of specialized pro-resolving mediators (SPMs), such as resolvins, protectins, and maresins, derived from EPA and DHA (González-Alva et al., 2024; Savran and Sağlam, 2024). Also, CLO has been shown to be active against *P. gingivalis* by inhibiting dipeptidyl peptidases (DPP). It also interferes with nucleic acid synthesis and central metabolic pathways, beginning with amino acid metabolism (Murai et al., 2024). Similarly, caffeine has also demonstrated an antibacterial effect against *P. gingivalis* at concentrations of 1%, 1.25%, 1.5%, and 3% (Sari et al., 2023). This effect has been attributed to its ability to alter the structural integrity of the bacterial cell membrane, interfering with its vital functions and potentially inducing lysis (Kim et al., 2016; Sari et al., 2023). Among the studies analyzed (Sari et al., 2023; González-Alva et al., 2024; Savran and Sağlam, 2024), we must again be cautious with their interpretation given that their methodological quality is moderate.

### Antioxidant activity

CLO has demonstrated an antioxidant effect through its ability to reduce ROS by increasing gluthatione, catalase, and superoxide dismutase, which protects against oxidative cell damage and could contribute to the inhibition of RANKL-induced osteoclastogenesis in periodontitis (Verma et al., 2016; Lima et al., 2017; Tanideh et al., 2020). By interfering with this activation, it could attenuate bone destruction induced by oxidative stress (Corrêa et al., 2017). This mechanism suggests that the antioxidant effect of curcumin not only limits tissue damage but could also promote the preservation of alveolar bone. Nevertheless, the quality of the aformentioned articles does not allow us to establish this conclusion with complete certainty, given the concerns regarding the methodological quality of the articles.

In the case of ω-3 PUFAs, prolonged use (6 months) has been observed in animal models to promote mitochondrial maintenance through biogenesis and autophagy mechanisms, suggesting a protective role against oxidative damage. Furthermore, resolvins and protectins derived from these lipids exhibit antioxidant properties that could reduce tissue damage associated with oxidative stress (González-Alva et al., 2024; Savran and Sağlam, 2024). Finally, caffeine, chlorogenic acid, and other compounds present in *Robusta coffee* beans have been linked to a decrease in ROS, which can interfere with intracellular signaling in bacteria and contribute to their death. Furthermore, this antioxidant effect could have beneficial implications for host tissues, helping to prevent oxidative damage associated with inflammation (Shushtari and Abtahi Froushani, 2017; Sari et al., 2023).

### Multi-scale autoregulatory networks in the BrSM model

Based on the discussion, a broad field of development is evident for the therapeutic use of natural molecules as adjuncts in the treatment of periodontitis. Although this review included a limited group of molecules, a wide variety of them exist that warrant further study. An example of this is boldine, an alkaloid present in *Peumus boldus*, which showed favorable results in the only study found that met the inclusion criteria, but due to the limited evidence, a more in-depth analysis was not possible (Cafferata et al., 2021). Also, rosmarinic acid and its components have recently arised as a potent immuno-modulator (Zdarilová et al., 2009; Kostić et al., 2017). This type of limitation highlights the need for continued research in this area.

Periodontitis occurs when the auto-regulatory networks that make up the individual are compromised (Goldman et al., 2015). People at risk of periodontitis encourage daily genetic, epigenetic, and environmental challenges that alter/modify their auto-regulating capacity. If these challenges occur daily such as the presence of keystone pathogens, smoking, poor oral hygiene, stress, and depression, among others, they can lead to the clinical manifestation of the disease. In recent years, periodontitis, through CLIP its low-grade inflammatory phenotype, has been linked to other chronic non-communicable diseases (Festa et al., 2002; López et al., 2002; López et al., 2005; Tonetti and Van Dyke, 2013; Teixeira et al., 2017; Cecoro et al., 2020). This implies that the concept of BrSM “common disease-state signature” is fully applicable (Goldman et al., 2015). In other words, many diseases share common functional pathways, suggesting that treatments and drugs may be more effective by targeting these biological networks rather than the pathogenic-causal approach. Thus, the BrSM proposed a therapeutic design based on mimic, modulate, or promote body’s auto-regulatory mechanisms. This does not imply replacing the gold standard therapy in periodontal treatment, but rather using auto-regulating molecules instead of traditional drugs that produce adverse effects. As a simple example, antibiotic therapy can profoundly disrupt the gut microbiota by reducing diversity and depleting beneficial bacteria within days. While some recovery occurs within weeks, full restoration of the initial microbial composition is slow and may take months to several years (Raymond et al., 2016; Bhalodi et al., 2019; Ng et al., 2019; Elvers et al., 2020; Cusumano et al., 2025; Shayista et al., 2025). In other words, a person who undergoes antibiotic treatment modifies their gut microbiota for life, potentially leading to increased susceptibility to other diseases. Nevertheless, this statement should be considered with caution, weighing the risks and benefits. Antibiotic prophylaxis is essential for high-risk patients such as those with prosthetic heart valves, history of infective endocarditis, or certain congenital heart diseases, before invasive periodontal treatments (Suda et al., 2018; Bakhsh et al., 2021). Also, adjunctive pharmacological treatments including systemic antibiotics and antiseptic agents are essential for managing severe periodontitis stages, like periodontitis stage IV grade C, by reducing bacterial load and inflammation (Werner et al., 2025). Thus, systemic antibiotics, specifically metronidazole and amoxicillin, act as effective, evidence-based complementary treatments to mechanical scaling and root planing (Kapoor et al., 2012).

Therefore, the presence of bioregulatory molecules and their potential use in people with periodontitis is relevant. These are molecules that are consumed daily through diet, but which, in other concentrations and routes of administration, promote auto-regulation. From a public health perspective, treatment with natural molecules, which are generally accessible and low-cost, represents a promising and sustainable strategy. In the long term, their incorporation into treatment protocols could promote a more biocompatible approach, reduce antibiotic use, and contribute to the development of preventive and personalized dentistry. Nowadays, only curcumin and ω-3 PUFAs have enough evidence to support it use as adjuvant in periodontal therapy. Taken together, this bioregulatory molecules have been shown to be able to decrease or prevent bone resorption caused by periodontitis, both in experimental animal studies and in clinical trials in humans. However, piperine, caffeine, and CLO needs more clinical trials to analyze their clinical effects on periodontal therapy.

## Data Availability

The original contributions presented in the study are included in the article/**Supplementary Material**, further inquiries can be directed to the corresponding author/s.
