# Supplementary material for: Bioregulatory periodontal medicine: could the molecular networks, inflammation and system biology modify the auto-regulatory capacity of the patients?
Source: Front Physiol. 2026 Sep 14;17:1799419. doi: 10.3389/fphys.2026.1799419 (PMC13616636; doi:10.3389/fphys.2026.1799419)
Supplement: Supplementary file 1 [file DataSheet1.pdf]

TABLE Nº2 Risk of bias of animal trials targeting bacteria in brain

	SYRCLE's Tool	*Study*<br>Tanideh et al, 2020	Lima et al, 2017	Alexandre et al, 2017	Sari et al, 2023	Dong et al, 2015	Guimaraes-Stabili et al, 2019	Hosadurga et al, 2014	Bakir et al, 2016	Correa et al, 2017	Akpinar et al, 2018	Xiao et al, 2018	Pimentel et al, 2020	Sha et al, 2020	Iova et al, 2021	Mohammad et al, 2022
1	**Was the allocation sequence adequately generated and applied?**	**Yes**	**Unclear**	**Unclear**	**Unclear**	**Yes**	**Unclear**	**Yes**	**Unclear**	**Yes**	**Yes**	**Yes**	**Unclear**	**Yes**	**Unclear**	**Unclear**
	*Did the investigators describe a random component in the sequence generation process such as: ■ Referring to a random number table; ■ Using a computer random number generator.	No	No	No	No	Yes	No	No	No	No	Unclear	No	No	No	No	No
2	**Were the groups similar at baseline or were they adjusted for confounders in the analysis?**	**Unclear**	**Unclear**	**Yes**	**Unclear**	**Yes**	**Unclear**	**Unclear**	**Unclear**	**Unclear**	**Yes**	**Unclear**	**Unclear**	**Unclear**	**Unclear**	**Unclear**
	*Was the distribution of relevant baseline characteristics balanced for the intervention and control groups?	Unclear	Unclear	Yes	Unclear	Yes	Unclear	Unclear	Unclear	Unclear	Yes	Unclear	Unclear	Unclear	No	Unclear
	*If relevant, did the investigators adequately adjust for unequal distribution of some relevant baseline characteristics in the analysis?	No	No	Unclear	No	No	Unclear	No	Unclear	No	No	Unclear	No	No	No	No
	*Was the timing of disease induction adequate?	Yes	Yes	Yes	Yes	Yes	Yes	Yes	Yes	Yes	Yes	Yes	Yes	Yes	Yes	Yes
3	**Was the allocation to the different groups adequately concealed during?**	**Unclear**	**Unclear**	**Unclear**	**No**	**Yes**	**Unclear**	**No**	**No**	**Unclear**	**Unclear**	**Unclear**	**No**	**Unclear**	**Unclear**	**No**
	*Could the investigator allocating the animals to intervention or control group not foresee assignment due to one of the following or equivalent methods? ■ Third-party coding of experimental and control group allocation central randomization by a third party ■ Sequentially numbered opaque, sealed envelopes	Unclear	Unclear	No	No	Yes	Unclear	No	No	No	No	No	No	Unclear	Unclear	No
4	**Were the animals randomly housed during the experiment?**	**Unclear**	**Unclear**	**Unclear**	**Unclear**	**Yes**	**Yes**	**Unclear**	**Unclear**	**Yes**	**Unclear**	**Unclear**	**Unclear**	**Unclear**	**Unclear**	**Unclear**
	*Did the authors randomly place the cages or animals within the animal room/facility? ■ Animals were selected at random during outcome assessment	Unclear	No	Unclear	No	Yes	Unclear	No	No	No	Unclear	Unclear	No	No	No	No
	*Is it unlikely that the outcome or the outcome measurement was influenced by not randomly housing the animals?	Unclear	Unclear	Unclear	Unclear	Unclear	Yes	Unclear	Unclear	Yes	Unclear	Unclear	Unclear	Unclear	Unclear	Unclear
5	**Were the caregivers and/or investigators blinded from knowledge which intervention each animal received during the experiment?**	**Unclear**	**Unclear**	**Yes**	**No**	**Unclear**	**Unclear**	**Unclear**	**Unclear**	**Unclear**	**Unclear**	**Unclear**	**Unclear**	**Unclear**	**No**	**Unclear**
	*Was blinding of caregivers and investigators ensured, and was it unlikely that their blinding could have been broken?	Unclear	Unclear	Yes	No	Unclear	No	Unclear	Unclear	Unclear	Unclear	Unclear	Unclear	Unclear	No	Unclear
6	**Were animals selected at random for outcome assessment?**	**No**	**Unclear**	**Unclear**	**Unclear**	**Yes**	**Yes**	**No**	**Unclear**	**Yes**	**Unclear**	**Unclear**	**No**	**No**	**No**	**No**
	*Did the investigators randomly pick an animal during outcome assessment, or did they use a random component in the sequence generation for outcome assessment?	No	No	Unclear	No	Unclear	Yes	No	No	Yes	Unclear	No	No	No	No	No
7	**Was the outcome assessor blinded?**	**Unclear**	**Yes**	**Yes**	**Yes**	**Yes**	**Yes**	**Unclear**	**Yes**	**Yes**	**Yes**	**Yes**	**Yes**	**Unclear**	**Unclear**	**Unclear**
	*Was blinding of the outcome assessor ensured, and was it unlikely that blinding could have been broken?	No	Unclear	Yes	No	Yes	Yes	No	Yes	Yes	Yes	No	Yes	No	No	No
	*Was the outcome assessor not blinded, but do review authors judge that the outcome is not likely to be influenced by lack of blinding?	Unclear	Not apply	No	Yes	Not apply	Not apply	Unclear	Not apply	Not apply	Not apply	Yes	Not apply	Unclear	Unclear	Unclear
8	**Were incomplete outcome data adequately addressed?**	**Unclear**	**Unclear**	**Yes**	**Unclear**	**Unclear**	**Unclear**	**Unclear**	**Unclear**	**Unclear**	**Unclear**	**Unclear**	**Unclear**	**Unclear**	**No**	**Unclear**
	*Were all animals included in the analysis?	Unclear	Unclear	Yes	Unclear	Yes	Unclear	Unclear	Unclear	Unclear	Yes	Unclear	Unclear	Yes	Unclear	Unclear
	*Were the reasons for missing outcome data unlikely to be related to true outcome? (e.g., technical failure)	Unclear	Unclear	Unclear	Unclear	Unclear	Yes	Unclear	Unclear	Unclear	Unclear	Unclear	Unclear	Unclear	Unclear	Unclear
	*Are missing outcome data balanced in numbers across intervention groups, with similar reasons for missing data across groups?	Unclear	Unclear	Unclear	Unclear	Unclear	Unclear	Unclear	Unclear	Unclear	Unclear	Unclear	Unclear	Unclear	No	Unclear
	*Are missing outcome data imputed using appropriate methods?	No	Unclear	Unclear	No	Unclear	No	No	No	No	No	No	No	No	No	No
9	**Are reports of the study free of selective outcome reporting?**	**Unclear**	**Unclear**	**Yes**	**Unclear**	**Yes**	**Yes**	**Unclear**	**Unclear**	**Yes**	**Unclear**	**Unclear**	**No**	**Yes**	**Unclear**	**Unclear**
	*Was the study protocol available and were all of the study's pre-specified primary and secondary outcomes reported in the current manuscript?	No	No	Unclear	No	No	Yes	No	No	No	Unclear	No	No	No	No	No
	*Was the study protocol not available, but was it clear that the published report included all expected outcomes (i.e. comparing methods and results section)?	Unclear	Unclear	Yes	Unclear	Yes	Unclear	Unclear	Unclear	Yes	No	Yes	No	Yes	Unclear	Unclear
10	**Was the study apparently free of other problems that could result in high ROB?**	**Unclear**	**Unclear**	**Yes**	**Unclear**	**Yes**	**Yes**	**Unclear**	**Unclear**	**Yes**	**Yes**	**Yes**	**Unclear**	**Unclear**	**Unclear**	**Unclear**
	*Was the study free of contamination (pooling drugs)?	Yes	Yes	Yes	Unclear	Yes	Yes	Yes	Yes	Yes	Yes	Yes	Yes	Yes	Yes	Yes
	*Was the study free of inappropriate influence of funders?	Yes	Yes	Yes	Yes	Unclear	Unclear	Yes	Ucleat	Yes	Yes	Yes	Unclear	Unclear	Yes	Yes
	*Was the study free of unit of analysis errors?	Unclear	Yes	Yes	Unclear	Yes	Yes	Unclear	Unclear	Yes	Yes	Yes	Yes	Yes	Unclear	Unclear
	*Were design-specific risks of bias absent?	Unclear	Unclear	Yes	Unclear	Yes	Yes	Unclear	No	Unclear	Yes	Yes	Unclear	Unclear	Unclear	Unclear
	*Were new animals added to the control and experimental groups to replace drop-outs from the original population?	No	No	No	No	No	No	No	No	No	No	No	No	No	No	No

* Item 1 was answered as Yes*, when random allocation was mentioned but no reference to a sequence generation process. In particular, allocation is often reffered to a third party generally associated to the maintenance of the animal facilty and not directly involved to the investigators or the project itself, hence allocation is 'random' for investigators. Item 2 was answered as Yes or Unclear, if two or more of the signalling questions were answered as Yes or Unclear, respectively. Item 4 was answered as Yes, if one of the signalling questions was answered as Yes, when one of the signalling questions was answer as No and the other as unclear then the principal item was rated as Unclear. Item 5 was answered as Yes*, when blinding from knowing which animals was in intervention/control groups was mentioned but there is no reference of how it was assured. Item 7 was answered as Yes, if one of the signalling questions was answered as Yes, when one of the signalling questions was answer as No and the other as unclear then the principal item was rated as Unclear. Item 8 was rated as Unclear, if two or more of the signalling questions were answered as Unclear, if there was a tie between Yes/No and Unclear among the signalling questions, the principal item was rated as Unclear. Item 9 was rated as Yes, if one of the signalling questions was rated as Yes. Item 10 was rated as Yes, if most of the signalling questions were answered as Yes.

TABL							
	Mohammad et al, 2023	Wang et al, 2023	Safitri et al, 2024	Jalal et al, 2019	Ozaki et al, 2020	Dogan et al, 2021	González-Alva et al, 2024
1	**Yes**	**Yes**	**Yes**	**Yes**	**Yes**	**Yes**	**Yes**
	No	Yes	No	No	No	No	Unclear
2	**Unclear**	**Unclear**	**Unclear**	**Unclear**	**Unclear**	**Yes**	**Yes**
	Unclear	Unclear	Unclear	Unclear	Unclear	Yes	Yes
	Unclear	No	Unclear	No	No	Unclear	No
	Yes	Yes	Yes	Yes	Yes	Yes	Yes
3	**No**	**Unclear**	**No**	**Unclear**	**Unclear**	**Unclear**	**No**
	No	Unclear	No	Unclear	No	Yes	No
4	**Unclear**	**Unclear**	**Unclear**	**Unclear**	**Yes**	**Unclear**	**Yes**
	No	No	No	Unclear	No	Unclear	Unclear
	Unclear	Unclear	Unclear	Unclear	Yes	Unclear	Yes
5	**No**	**Unclear**	**No**	**Unclear**	**No**	**Unclear**	**Unclear**
	No	Unclear	No	Unclear	No	Unclear	No
6	**No**	**Unclear**	**No**	**No**	**Yes**	**No**	**Yes**
	No	Unclear	No	No	Unclear	No	Yes
7	**Unclear**	**Unclear**	**Unclear**	**Unclear**	**Unclear**	**Yes**	**Unclear**
	No	No	No	No	No	Yes	No
	Unclear	Unclear	Unclear	Unclear	Unclear	Not apply	Unclear
8	**Unclear**	**Unclear**	**Unclear**	**Unclear**	**Unclear**	**Yes**	**Yes**
	Unclear	Yes	Unclear	Unclear	Unclear	Yes	Yes
	Unclear	Unclear	Unclear	Unclear	Unclear	Yes	Yes
	Unclear	No	Unclear	No	Unclear	Yes	Yes
	No	No	No	No	No	No	No
9	**No**	**Unclear**	**Unclear**	**Unclear**	**Unclear**	**Yes**	**Yes**
	No	No	No	No	No	Unclear	Yes
	Unclear	Yes	Unclear	Unclear	Unclear	Yes	No
10	**Unclear**	**Unclear**	**Unclear**	**Unclear**	**Yes**	**Yes**	**Yes**
	Unclear	Yes	Yes	Yes	Yes	Yes	Yes
	Yes	Yes	Yes	Unclear	Yes	Yes	Yes
	Unclear	Yes	Unclear	Yes	Yes	Yes	Yes
	No	Unclear	Unclear	No	Unclear	Yes	Yes
	No	No	No	No	No	No	No
