# Supplementary material for: Bioregulatory periodontal medicine: could the molecular networks, inflammation and system biology modify the auto-regulatory capacity of the patients?
Source: Front Physiol. 2026 Sep 14;17:1799419. doi: 10.3389/fphys.2026.1799419 (PMC13616636; doi:10.3389/fphys.2026.1799419)
Supplement: Supplementary file 2 [file Table2.docx]

**SUPPLEMENTAL FILE 2** Search strategies

Search Strategies for MEDLINE, EMBASE, WEB OF SCIENCE and LILACS databases were the following:

**MEDLINE**

(((((((((((Periodontitis [MeSH Terms]) OR Boldine[MeSH Terms]) OR Curcumin[MeSH Terms]) OR Calendula[MeSH Terms]) OR Caffeine[MeSH Terms]) OR Piperine[MeSH Terms]) OR Omega-3 fatty acids[MeSH Terms]) OR Experimental periodontitis[MeSH Terms]) OR Ligature-induced[MeSH Terms]) OR oral bacteria[MeSH Terms]) OR oral microbiota[MeSH Terms])AND ((((((((periodontitis[MeSH Terms]) OR chronic periodontitis[MeSH Terms]) OR experimental periodontitis) OR ligature periodontitis) OR oral gavage periodontitis[MeSH Terms]) OR experimental periodontitis model) OR bone resorption[MeSH Terms]) OR periodontal bone resorption) OR bone destruction[MeSH Terms]).

**EMBASE**

('periodontitis'/exp OR ‘periodontitis model'/exp OR 't oral gavage'/exp OR ‘ligature method'/exp OR ‘bone resorption'/exp OR ‘bone destruction'/exp OR 'inflammation' OR ‘periodontal bacteria'/exp OR ‘periodontal pathogen'/exp OR ‘oral bacteria' OR ‘oral pathogen' OR ‘keystone pathogen' OR 'pathobiont' OR 'microbiota' OR 'dysbiosis' OR ‘dysbiotic microbiota'/exp OR ‘subgingival microbiota' OR ‘periodontal microbiota' OR ‘bacteria detection'/exp OR ‘bacteria quantification' OR ‘bacteria identification') AND ('boldine'/exp OR 'curcumin'/exp OR ‘calendula'/exp OR ‘caffeine'/exp OR 'Piperine'/exp OR ‘rosmarinic acid'/exp OR ‘omega-3 fatty acids‘/exp OR ‘omega-6 fatty acids‘/exp OR ‘Alzheimer’s disease'/exp) AND [embase]/lim

**WEB OF SCIENCE**

#1	TS=(Periodontitis OR Pathogen OR Experimental periodontitis OR Bacteria OR keystone OR pathobiont)

#2	TS=(boldine OR curcumin OR calendula OR caffeine OR piperine OR Omega-3 fatty acids)

#3	#1 AND #2}

**LILACS**

“PERIODONTITIS EXPERIMENTAL" OR “BIOREGULACIÓN" [Palabras] or “BIOREGULADORES" [Palabras] and ( "PERIODONTITIS" ) or “EXPERIMENTAL PERIODONTITIS" [Palabras]
